# Aging-related olfactory loss is associated with olfactory stem cell transcriptional alterations in humans

**DOI:** 10.1172/JCI155506

**Published:** 2022-02-15

**Authors:** Allison D. Oliva, Rupali Gupta, Khalil Issa, Ralph Abi Hachem, David W. Jang, Sebastian A. Wellford, E. Ashley Moseman, Hiroaki Matsunami, Bradley J. Goldstein

**Affiliations:** 1 Department of Head and Neck Surgery & Communication Sciences and; 2 Department of Immunology,; 3 Department of Molecular Genetics and Microbiology,; 4 Duke Institute for Brain Sciences,; 5 Department of Neurobiology, and; 6 Center for the Study of Aging and Human Development, Duke University School of Medicine, Durham, North Carolina, USA.

**Keywords:** Aging, Neuroscience, Adult stem cells, Neuronal stem cells

## Abstract

**BACKGROUND:**

Presbyosmia, or aging-related olfactory loss, occurs in a majority of humans over age 65 years, yet remains poorly understood, with no specific treatment options. The olfactory epithelium (OE) is the peripheral organ for olfaction and is subject to acquired damage, suggesting a likely site of pathology in aging. Adult stem cells reconstitute the neuroepithelium in response to cell loss under normal conditions. In aged OE, patches of respiratory-like metaplasia have been observed histologically, consistent with a failure in normal neuroepithelial homeostasis.

**Methods:**

Accordingly, we have focused on identifying cellular and molecular changes in presbyosmic OE. The study combined psychophysical testing with olfactory mucosa biopsy analysis, single-cell RNA-Sequencing (scRNA-Seq), and culture studies.

**Results:**

We identified evidence for inflammation-associated changes in the OE stem cells of presbyosmic patients. The presbyosmic basal stem cells exhibited increased expression of genes involved in response to cytokines or stress or the regulation of proliferation and differentiation. Using a culture model, we found that cytokine exposure drove increased TP63, a transcription factor acting to prevent OE stem cell differentiation.

**Conclusions:**

Our data suggest aging-related inflammatory changes in OE stem cells may contribute to presbyosmia via the disruption of normal epithelial homeostasis. OE stem cells may represent a therapeutic target for restoration of olfaction.

**Funding:**

NIH grants DC018371, NS121067, DC016224; Office of Physician-Scientist Development, Burroughs-Wellcome Fund Research Fellowship for Medical Students Award, Duke University School of Medicine.

## Graphical abstract

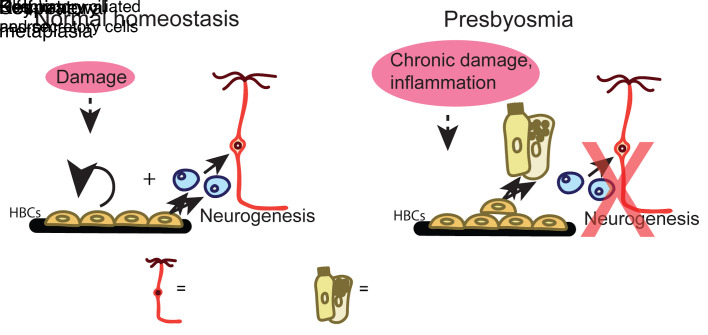

## Introduction

The olfactory system detects volatile odors, providing chemosensory input via the first cranial nerve. Olfaction contributes to daily social interactions and quality of life, is important in the detection of smoke or dangerous chemicals, and underlies flavor perception in combination with gustatory input from taste buds. Loss of olfaction can result in depression, nutritional disorders, and increased mortality (1–3). Acquired olfactory loss, termed anosmia, can occur due to trauma, viral infections, including COVID-19, sinonasal disease, such as sinusitis, or neurodegenerative conditions, such as Alzheimer disease (4–8). In addition, aging-related loss of smell (presbyosmia) has been well described, but remains poorly understood. Population studies using psychophysical testing have identified half of the subjects over age 65 years and greater than two-thirds of those over the age of 80 as exhibiting impaired olfaction (9, 10). However, there are no effective therapies for presbyosmia. To facilitate development of specific treatments, an understanding of the mechanisms underlying this condition is required.

Limited to the olfactory cleft, the olfactory epithelium (OE) is a true neuroepithelium containing bipolar chemosensory neurons, while the remainder of the nasal cavity is lined by respiratory epithelium consisting of ciliated cells, secretory cells, and basal cells. The OE contains neurogenic populations known as globose basal cells (GBCs) and horizontal basal cells (HBCs) (11). In rodent models, during normal olfactory sensory neuron turnover, the GBCs divide and differentiate to produce new neurons, while the HBCs are relatively quiescent (12–14). However, when faced with injuries that disrupt the sustentacular cell layer of the OE, the reserve HBC stem cells are activated and serve as the main source of olfactory sensory neurogenesis (15–18). Human OE is exposed continuously to potential damage in the form of viral or other infections, inflammation driven by allergens and other inspired irritants, or trauma. Thus, the proper function of the neurogenic cycle is critical for homeostasis within the OE and, in turn, a functioning sense of smell.

**Figure 1:**
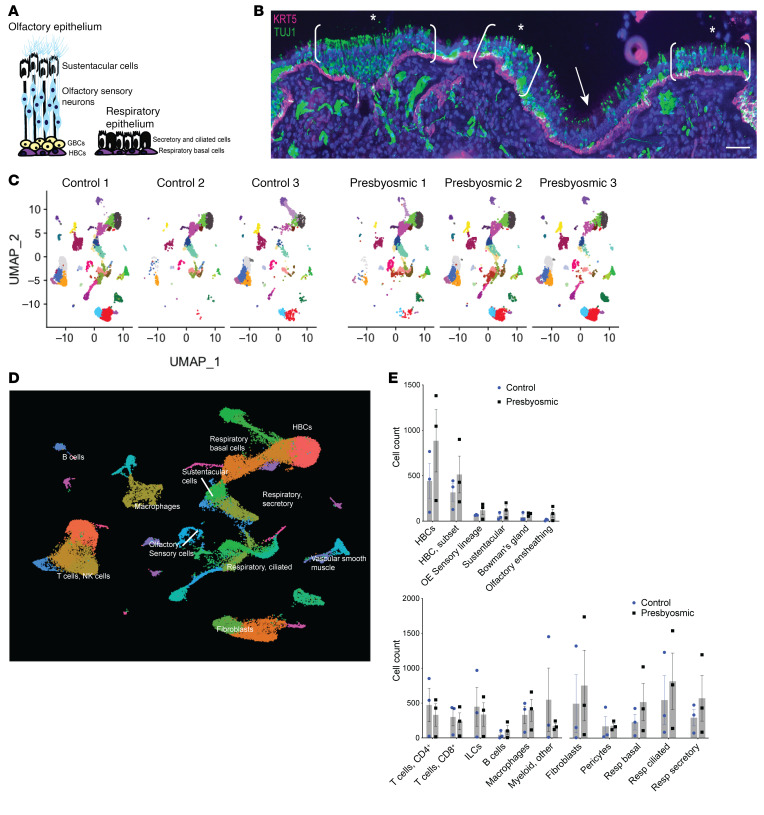
Integrated analysis of 36,091 cells from human olfactory epithelial biopsies of normosmic and presbyosmic adults. (**A**) Schematic illustration of cell types in human olfactory and respiratory nasal epithelium. (**B**) Human OE from an 80-year-old presbyosmic subject; brackets and asterisks indicate areas containing TUJ1^+^ OE neurons (green); a patch of aneuronal respiratory-like metaplasia is marked by the white arrow; HBCs are labeled with anti-KRT5 (magenta). Nuclei are stained with DAPI (blue). Scale bar: 50 μM. (**C**) UMAP projection showing integrated data set split into separate plots by sample. (**D**) UMAP projection of combined data set; clusters are annotated according to canonical markers. (**E**) Contributions from normosmic or presbyosmic cohorts are quantified for indicated cell categories. Data are represented as mean ± SEM. *n* = 3 samples per cohort.

Consistent with rodent models, neurogenic basal cells are active in the OE of adult humans (19, 20). Thus, one potential explanation for presbyosmia is an aging-related process of neurogenic exhaustion. In this model, aged adult neurogenic cell populations are believed to be exhausted and either no longer present or, if present, incapable of reconstituting the neuronal populations (21). As a result, as neurons are depleted with normal ongoing damage or “wear-and-tear,” the ability to maintain an intact OE is lost. Histologic study of olfactory biopsies or autopsy samples demonstrating areas of respiratory-like metaplasia in aged adults (20–22) supports the idea that the neuronal layer can be depleted, leaving an aneuronal, respiratory-like barrier epithelium in its place (Figure 1, A and B). However, adult olfactory neurogenesis is a highly regulated process involving a complex cellular milieu including immune cells, supporting cell populations, ensheathing glia, stromal and vascular cells, and multiple populations of basal stem and progenitor pools (23). While histologic findings have been described, direct evidence for molecular alterations in neurogenic OE populations in presbyosmic humans is lacking, limiting the ability to identify new treatment strategies for aging-related olfactory loss.

Classic fate-mapping studies following rodent OE damage defined HBCs as the multipotent OE reserve stem cell, activated by severe injury (15). Of interest, an experimental model of severe chronic olfactory inflammation in mice involving OE exposure to TNF-α showed that HBCs may adopt a protective “barrier defense” transcriptional program, driven by NF-κB or JNK signaling (24). In this model, prolonged inflammation prevents stem cell neurodifferentiation despite neuron depletion and increased signaling between HBCs and immune cells (25). Together, the data from rodent olfactory injury-regeneration studies and the potential influence of inflammatory signaling suggest a model in which some human olfactory disorders, such as presbyosmia, may involve OE stem cell derangements linked to local microenvironmental cues.

Accordingly, the aim of this study is to investigate transcriptional changes in presbyosmic adult human OE samples at single-cell resolution. The single-cell approach is useful for identifying unbiased gene expression patterns in highly heterogenous tissues and enabling differential gene expression (DE) analysis between subpopulations of interest. We hypothesized that olfactory basal stem cells in older adults with hyposmia may exhibit gene expression changes in pathways involved with neurogenic activity compared with normosmic controls. To test this hypothesis, we performed single-cell RNA-Seq (scRNA-Seq) analysis of human OE biopsies from normosmic control subjects and hyposmic subjects aged 65 years or older as well as culture and immunohistochemical assays. All subjects included in our study underwent psychophysical olfactory function testing using the Smell Identification Test (SIT) (Sensonics Inc.) to measure their olfactory ability.

## Results

### Study cohort.

To investigate mechanisms involved with presbyosmia, we obtained olfactory mucosa biopsies from 8 adult human subjects, ages 51 to 80 years, 4 male and 4 female (demographic details provided in Table 1). We sought controls with normosmic function and therefore included 1 subject slightly below age 65 to meet these criteria, but otherwise avoided inclusion of young samples. SIT scores greater than 33 are considered normosmic; 30 to 33, mildly hyposmic; 26 to 29, moderately hyposmic; 19 to 25, severely hyposmic; and 6 to 18, anosmic (with slight adjustments for age/sex). We processed samples for scRNA-Seq from 6 subjects. One cohort consisted of 3 normosmic subjects, based on psychophysical olfactory assessment, with SIT scores of 34 to 37; the other cohort consisted of presbyosmic subjects, with SIT scores 11 to 29 and age greater than 65 years. Active rhinitis or sinusitis was excluded by nasal endoscopy and/or noncontrast sinus CT scan showing absence of nasal polyps, severe edema or purulence, or history. The diagnosis of presbyosmia was established based on SIT score and absence of other obvious cause for sensorineural hyposmia, such as prior head trauma, postviral olfactory disorder, or neurodegenerative disease. Biopsies were obtained from the olfactory cleft at the time of transnasal endoscopic surgery for access to the sphenoid or skull base that was unrelated to nasal or olfactory disorders (26).

### Epithelial analysis.

For olfactory mucosa biopsies, we generated high-quality single-cell transcriptional profiles for 36,091 cells. Despite being obtained from the superior posterior septum, typically lined by surface OE rather than sinonasal surface respiratory epithelium (RE), biopsies comprised mixtures of both OE and RE cells (Figure 1, A–C). Mixed epithelial populations were not unexpected, since there is a well-documented patchy replacement of OE by respiratory-like surface epithelium in adult humans (20, 22). An example of discontinuous intact neuroepithelium with aneuronal patches is shown in a presbyosmic biopsy stained to visualize olfactory neurons with antibody Tuj1, recognizing neuron-specific β-tubulin (Figure 1B). Using unsupervised clustering of scRNA-Seq data, we visualized cellular composition in uniform manifold approximation projection (UMAP) plots; distinct populations were annotated as we and others have described, based on known marker gene expression (19, 27, 28). Clustering was visualized for individual biopsies (Figure 1C) and in an integrated and batch-corrected combined plot (Figure 1D). Because we used full-thickness surgical punch-style biopsies rather than a brush biopsy technique, our samples captured surface epithelial cells as well as large numbers of stromal cells, vascular cells, and immune populations (Figure 1, D and E). While one presbyosmic subject with total anosmia had nearly no olfactory neurons present, all other samples contained sensory cell populations, ranging from 63 to 186 cells per sample (Figure 1, C–E, and Supplemental Figure 1; supplemental material available online with this article; https://doi.org/10.1172/JCI155506DS1). Neuron lineage cluster analysis identified GBCs and immature and mature olfactory neurons and identified expression of olfactory receptor transcripts, 56 of which were not found in our previous scRNA-Seq data set (Supplemental Figure 1, Supplemental Tables 2–6). The relative cellular composition is indicated for presbyosmic and normosmic cohorts (Figure 1E). DotPlot visualization of transcripts highly enriched in specific cell clusters provides an overview of annotated cell phenotypes (Supplemental Figure 2). Initial analysis demonstrated that our samples capture the range of cell populations present in olfactory mucosa. While this approach provides limited ability to draw definitive conclusions about relative numbers of specific cell populations, single-cell profiling is particularly well suited to exploring transcriptional changes focused on identical cell populations from diseased versus control cohorts via differential expression analysis (Supplemental Figure 3).

### Focused cell cluster analysis: olfactory stem cells.

**Figure 2:**
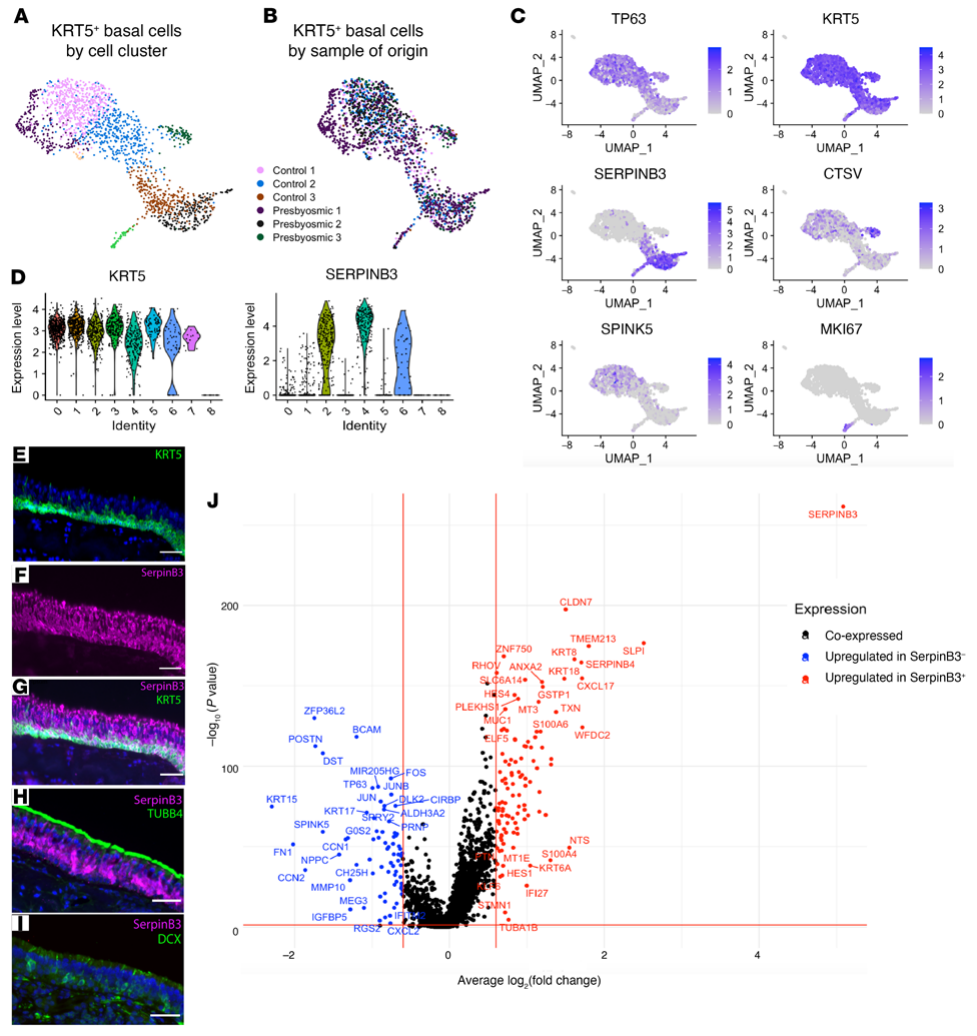
Basal cell transcriptional heterogeneity. (**A**) UMAP plot visualizing the KRT5^+^ subset from integrated data set after unbiased reclustering. (**B**) Basal cell subset identifying cells by sample of origin; note substantial overlap of sample contributions. (**C**) Selected gene expression plots demonstrating heterogeneity among basal cells. (**D**) Comparison of KRT5 and SERPINB3 expression across basal cell subclusters. (**E**–**G**) Human respiratory epithelium immunohistochemistry with SERPINB3 (magenta) expression in KRT5^+^ basal cells and the columnar or secretory cells. (**H**) Respiratory epithelium with colocalization of TUBB4 protein (green) apically and SERPINB3 (magenta). (**I**) OE from a normosmic subject costained for immature neuronal marker DCX (green) and SERPINB3 (magenta), absent. (**J**) Differential expression between the SERPINB3^+^ and SERPINB3^–^ basal cells. Genes significantly upregulated (*P* < 0.05 and log fold change >0.60) in SERPINB3^+^ cells are colored in red; genes significantly upregulated in SERPINB3^–^ cells are colored in blue. Nuclei in **E**–**I** are stained with DAPI (blue). Scale bars: 50 μM.

Because a patchy replacement of neuroepithelium with respiratory-like metaplastic surface epithelium, lacking neurons, is a prominent feature of presbyosmic biopsies (Figure 1B), a process of “neurogenic exhaustion” has been proposed as an underlying mechanism (22, 29). In this model, olfactory basal stem or progenitor cell populations would exhaust or change over time, resulting in an inability to support ongoing epithelial renewal. Accordingly, we focused attention on the stem cell clusters in presbyosmic versus control normosmic samples (Figure 2). The HBC, defined by KRT5 and TP63 expression, functions as the reserve stem cell population in the OE (15), and a biochemically similar basal cell also supports self-renewal of the respiratory epithelium (28). Replotting the KRT5^+^ basal cell cluster subset and visualizing sample contributions, we found that olfactory stem cells (HBCs) were abundant in presbyosmic biopsies (Figure 2, A and B). Nine subclusters of KRT5^+^ basal cells (which included respiratory and olfactory basal cells) were identified and further analyzed and included more than 4000 cells, with contributions from all biopsy samples (Figure 2A). For clarity, we refer to KRT5^+^ basal cells as HBCs only when they are associated with OE, and if they are from the respiratory epithelium, we refer to them as respiratory basal cells.

### Basal cell heterogeneity.

We identified here heterogeneity among the TP63^+^KRT5^+^ basal cells (Figure 2C). For instance, SERPINB3 strongly segregated to a subset of the basal cells present in our samples, as visualized in gene expression plots or violin plots (Figure 2, C and D). In our prior scRNA-Seq data set, SERPINB3 appeared to be enriched in respiratory rather than olfactory basal cells (19); here, immunohistochemical staining confirmed at the protein level that SERPINB3 colocalized in many KRT5^+^ basal cells in portions of surface epithelium *lacking* olfactory neurons, but coexpressing known respiratory epithelial markers such as TUBB4 (also referred to as β4-tubulin; ref. 29 and Figure 2, E–I). SERPINB3 was also present in more mature respiratory secretory cells (Figure 2, F and G). However, observed areas harboring olfactory neurons, labeled with neuron-specific markers such as DCX, lacked SERPINB3 (Figure 2I). Thus, SERPINB3 may be useful in distinguishing olfactory from respiratory cells in heterogenous samples. DE analysis comparing SERPINB3^+^ versus SERPINB3^–^ basal cells identified several genes that were significantly (*P* < 0.05, log_2_FC > 0.60) upregulated in SERPINB3^+^ basal cells (Figure 2J). Some of these enriched genes, such as HES4 and MUC1, have been demonstrated in respiratory cell lineages (28), and genes such as KRT8 and KRT18 are established nonneuronal markers in murine models (30). In contrast, transcripts that were enriched in SERPINB3^–^ basal cells, including KRT15, MEG3, CTSV, and SPINK5, are less well-studied. Visualizing SPINK5 and CTSV in UMAP plots, both transcripts localized broadly among the putative olfactory HBC clusters, although a small highly CTSV-enriched subset was identifiable (Figure 2C). CTSV is of potential interest, as it is a druggable cysteine protease with restricted CNS expression, thought to have roles in regulation of cell proliferation, adhesion, and, importantly, immune response (31, 32). SPINK5 encodes a proteinase inhibitor, also present in epidermal basal layers, with activity regulating proliferation via the Wnt pathway (33, 34); a role in HBCs has not been described. Based on these findings, our analysis in the remainder of this manuscript uses the term HBC in reference to the SERPINB3^–^ basal cell clusters in an effort to include only olfactory basal stem cells and exclude likely respiratory basal cells.

### Presbyosmic versus control stem cells.

**Figure 3:**
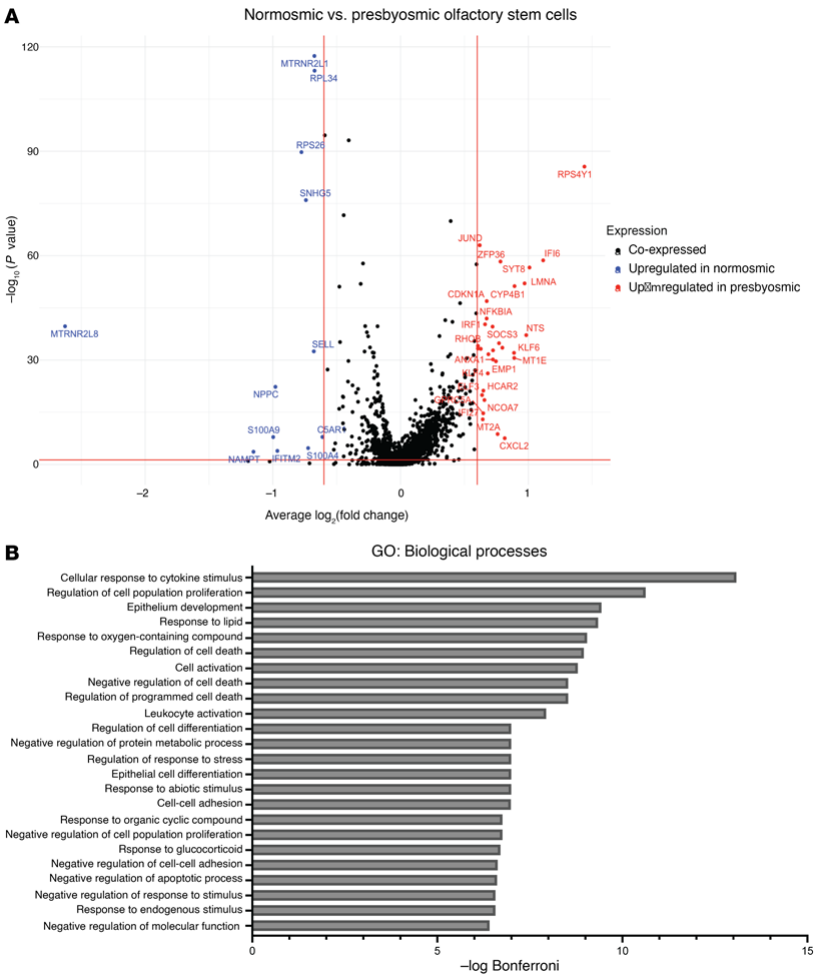
DE identifies presbyosmic stem cell dysfunction. (**A**) DE analysis between presbyosmic and normosmic HBCs; transcripts significantly upregulated (*P* < 0.05 and log fold change >0.60) in presbyosmic HBCs (red) and transcripts upregulated in normosmic HBCs (blue) are labeled. (**B**) GSEA of presbyosmic HBC-enriched transcripts, plotting the top significant biological process terms; inflammatory or injury response terms suggest HBC functional alterations.

We also performed DE analysis comparing HBCs from presbyosmic versus control groups (Figure 3 and Supplemental Tables 2–6). Our analysis identified 31 transcripts significantly upregulated in presbyosmic HBCs compared with normosmic control HBCs, including cytokine-response genes IFI6, IFI27, IRF1, KLF4, and KLF6 and anti-proliferative gene RHOB (Figure 3A and refs. 35–37). Gene set enrichment analysis (GSEA) of the upregulated set suggested strongly that the presbyosmic HBCs exhibit changes consistent with chronic inflammation. GSEA biological process categories included the processes cellular response to cytokine stimulus, regulation of cell proliferation, regulation of cell differentiation, and numerous terms relating to regulation of apoptosis or cell death (Figure 3B). Of interest, our findings from presbyosmic human HBCs are consistent with basal cell alterations identified in a mouse model of chronic local cytokine overexpression (25), providing support for a model of stem cell dysfunction in aging-related human olfactory loss. To exclude the possibility that HBC gene expression changes were driven by the sole totally anosmic sample in the presbyosmia group, we performed a reanalysis excluding this sample (Supplemental Figure 4) and identified similar HBC alterations.

### Immune landscape in presbyosmic olfactory mucosa.

**Figure 4:**
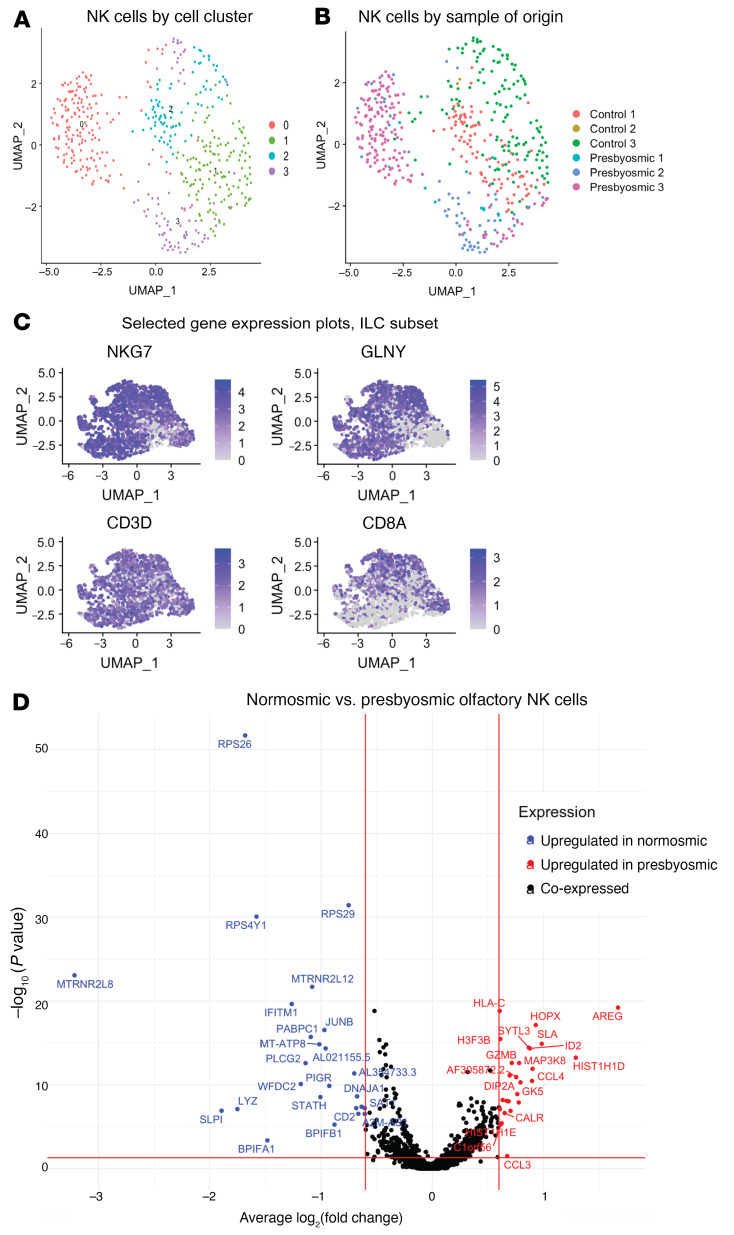
Gene expression changes in select presbyosmic immune cells. (**A**) A UMAP plot of the subset of NK cells after reclustering. (**B**) Sample origins are depicted. (**C**) Gene expression plots for selected innate lymphocyte compartment (NK/ILC) transcripts, (NKG7, GNLY, CD3D and CD8A); additional markers are depicted in Supplemental Figure 3. (**D**) DE between presbyosmic and normosmic immune cells; transcripts significantly upregulated (*P* < 0.05 and log fold change >0.60) in presbyosmic cells are colored in red, while transcripts significantly upregulated in normosmic are colored in blue.

Because we identified changes suggesting inflammatory responses in presbyosmic HBCs, we next explored the immune cells captured in olfactory biopsies. Focusing attention on immune cell populations within our integrated scRNA-Seq data set, we investigated DE between the presbyosmic and normosmic samples. CD8^+^ T cells, CD4^+^ T cells, innate lymphocytes (NK, NKT and innate lymphoid cells [ILCs]), CD68^+^ macrophages, and mast cells were identified (Supplemental Figure 5). Initial analysis suggested possible differences among the innate lymphocyte compartment (NK/ILC) cells from presbyosmic and normosmic control biopsies, and these were reclustered and visualized (Figure 4, A and B). While canonical “NK” markers NKG7 and GNLY were expressed uniformly (Figure 4C), DE analysis identified several upregulated genes in cells from presbyosmic subjects (Figure 4D). The most significantly upregulated gene was amphiregulin (AREG). AREG has been found to increase regulatory T cell proliferation and to protect and maintain epithelial stem cells in corneal epithelial injury models (38, 39). But perhaps most intriguingly, a subset of ILCs, ILC2s, are known to secrete AREG to bolster mucosal epithelial repair. AREG is a ligand for the EGF receptor, which is strongly expressed by olfactory HBCs, providing a mechanism of action to influence presbyosmic HBC activity (38). Indeed, ILC2-derived amphiregulin has been shown to play a prominent role in lung epithelial regeneration (40). Other genes upregulated in presbyosmic patients (Figure 4D), including H3F3B, SYTL3, and HOPX, are also associated with innate lymphocytes (41, 42). Likewise, we identified the C-C motif chemokine ligands CCL3 and CCL4 as enriched in presbyosmic cells, and chemokine signaling between immune cells and HBCs has been described in rodent models (25). Both CCL3 and CCL4 are reportedly expressed by subgroups of innate and adaptive lymphocytes, suggesting multiple lymphocyte subsets may actually contribute to HBC dysfunction. These data point to potential mechanisms for innate lymphocyte-derived proepithelial signals to influence olfactory versus respiratory replacement within the olfactory cleft.

### Lymphocyte–stem cell interactions in presbyosmia.

**Figure 5:**
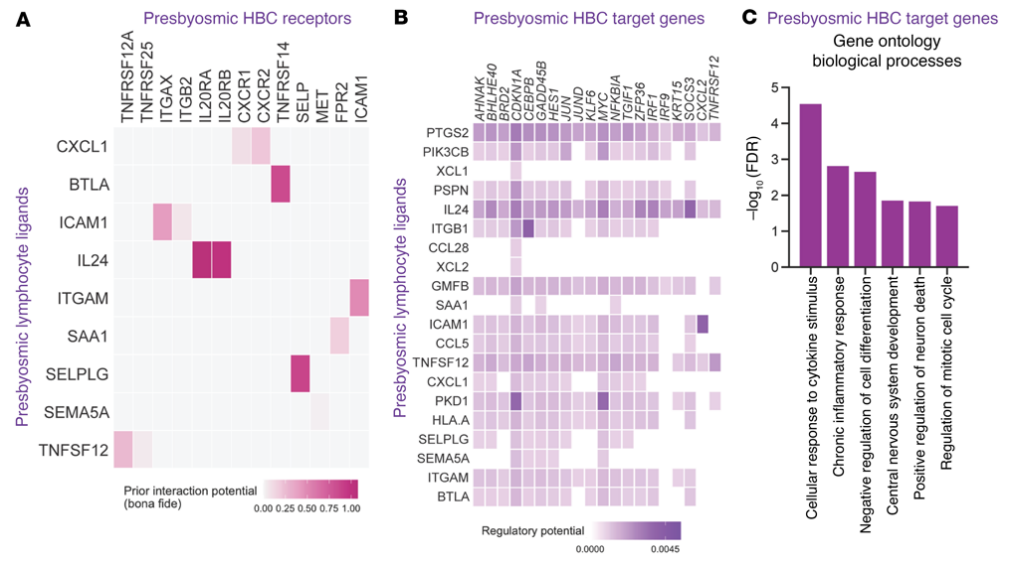
Presbyosmic immune cell subsets drive DE in stem cells. (**A**) NicheNet intercellular communication analysis identified receptors specifically upregulated in presbyosmic HBCs. These receptors are plotted with highly validated ligands expressed by presbyosmic NK, NKT, and CD8^+^ T cells. (**B**) Ligand-target matrix denoting the regulatory potential between presbyosmic NK, NKT, and CD8^+^ T cell ligands and selected target genes from the presbyosmic HBC program. (**C**) GSEA of presbyosmic HBC-enriched target genes identified by NicheNet analysis, plotting the selected biological process terms.

To explore potential lymphocyte interactions active in presbyosmic olfactory mucosa, we performed NicheNet ligand-receptor analysis (Figure 5), identifying potential pairings between receptors upregulated in presbyosmic HBCs and ligands expressed by presbyosmic NK, NKT, and CD8^+^ T cell subsets (43). A number of lymphocyte/HBC ligand pairings were enriched in our presbyosmic data sets (Supplemental Figure 6A), including inflammatory chemokines (CXCL1) and TNF family member TNFSF12 (TWEAK; Figure 5A). Lymphocyte-expressed IL-24 emerged as a potent interacting ligand with presbyosmic HBCs, expressing high levels of receptors IL-20RA and IL-20RB (Figure 5A and ref. 44). NK and T cells have been found to produce IL-24 (45–47), driving proinflammatory and allergic functions (48–50) and enhancing wound chronicity (51). The signaling pathways acting downstream of these receptor/ligand pairs included multiple target genes that we identified as significantly upregulated in presbyosmic HBCs (Figure 2J, Figure 5B, and Supplemental Figure 6, B and C), including CDKN1A (p21), a downstream target of all identified lymphocyte ligands. CDKN1A is of particular interest, as it can inhibit cell-cycle progression in response to DNA stress, and removal of CDKN1a enhances regenerative potential of hematopoietic or intestinal progenitors, while overexpression impairs wound healing (52, 53). Together, these findings suggest the intriguing hypothesis that, due to a persistent inflammatory milieu, CDKN1a activation in presbyosmic HBCs may contribute to impaired regenerative function. Other ligands of interest identified here included COX2 (PTGS2), associated with widespread potential activation of presbyosmic HBCs (Figure 5B). Overall, ligand/receptor target gene analysis revealed a signature of chronic inflammatory cytokine signaling and negative impact on cellular differentiation (Figure 5C and Supplemental Figure 6B), identifying potential mechanisms by which lymphocyte populations may negatively affect HBC function in presbyosmia.

### Other cell populations.

The other basal cell population in the OE, GBCs, were also evaluated. In rodent models, GBCs generally function as active neuronal progenitors, dividing and differentiating into olfactory neurons. Human OE typically contains sparse numbers of GBCs when probed by immunohistochemistry or as appears in published scRNA-Seq data (19, 20), likely due to the transient GBC state as a rapidly differentiating progenitor produced only as needed in response to neuronal loss. Here, a small pool of GBCs (42 cells) from presbyosmic and normosmic samples were analyzed (Supplemental Figure 7). While these cells exhibit expression of established GBC markers, including basic helix-loop-helix neurogenic transcription factors, analysis did show that inflammatory and early response genes were among upregulated DE transcripts in presbyosmic cells, including IER2, CXCR4, and JUNB. However, significant Gene Ontology (GO) terms for the set of upregulated presbyosmic GBC genes were not clearly informative, suggesting that the GBC phenotype is not substantially altered. These findings suggest that presbyosmic GBCs likely continue to function as transient neuronal precursors based on their transcriptional profiles.

### HBC culture model.

**Figure 6:**
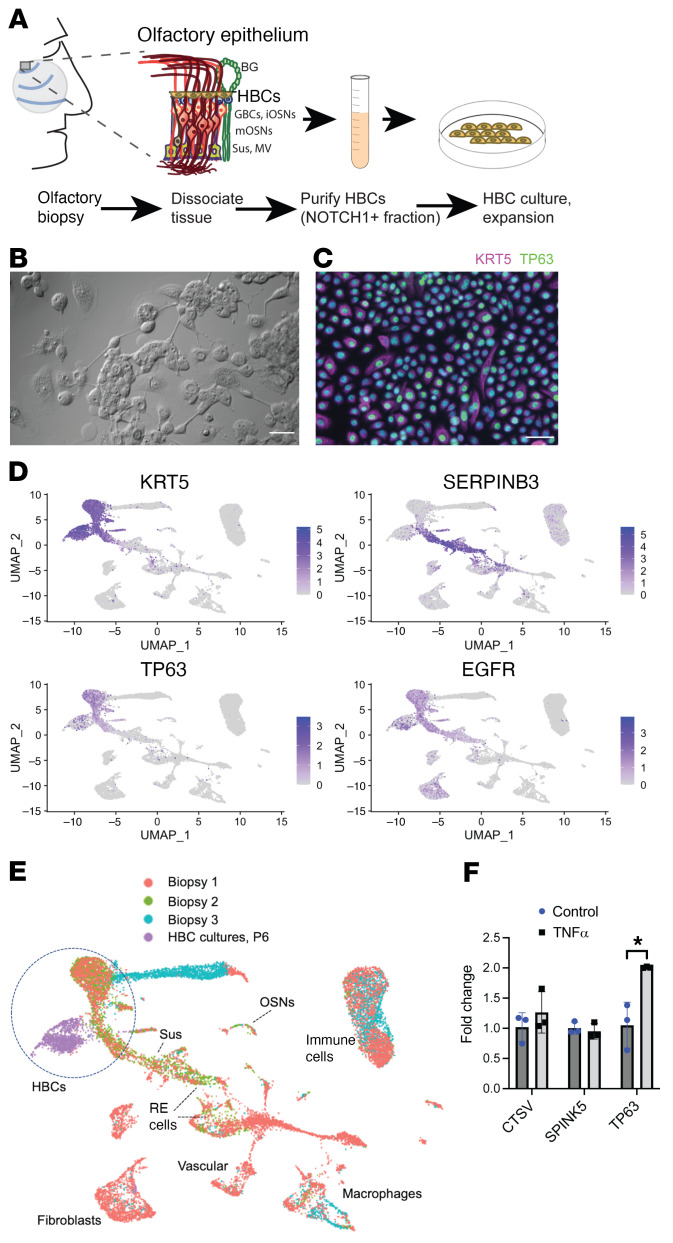
A cell culture model for human HBC analysis. (**A**) Experimental approach; human olfactory mucosa biopsy is dissociated, followed by immunoselection for NOTCH1^+^ cells, seeded onto laminin for in vitro expansion. (**B**) Differential interference contrast (DIC) view of typical adherent cell islands and (**C**) immunostaining of cultures showing near-uniform TP63^+^KRT5^+^ phenotype. (**D** and **E**) scRNA-Seq analysis of passage 6 HBC cultures; the culture single-cell data set was integrated with 3 control acutely harvested human olfactory biopsy scRNA-Seq data sets. FeaturePlots in **D** show gene expression of selected transcripts, and UMAP (**E**) showing sample origins indicates that culture-expanded cells (purple) cluster adjacent to acutely harvested olfactory HBCs, indicating transcriptional similarity, and largely lack SERPINB3. iOSNs, immature olfactory sensory neurons; mOSNs, mature neurons; BG, Bowman’s gland; MV, microvillar cells; Sus, sustentacular. (**F**) RT-qPCR assay of TNF-α–treated HBC cultures; note 2 fold-change increase in TP63 expression relative to controls (*P* = 0.04, *n* = 3). Scale bars: 50 μM.

To facilitate further mechanistic study of human olfactory stem cells, we sought to develop a human olfactory HBC culture model. We reasoned that immunoselection for cells expressing NOTCH1 from cell suspensions of human olfactory mucosa biopsies would enrich samples highly for HBCs, as the NOTCH1 surface receptor has been demonstrated on HBCs (18). Thus, we prepared a surgical olfactory mucosal biopsy sample from a normosmic subject for scRNA-Seq immediately following tissue harvest, and the remaining cell suspension was then NOTCH1 immunoselected for culture (Figure 6A). The NOTCH1^+^ fraction was maintained on 5% laminin in a well-established respiratory basal cell-growth medium for several passages. Importantly, the scRNA-Seq analysis of the portion of the biopsy used to establish this culture revealed almost exclusively olfactory rather than respiratory populations (see Figure 1, C and D). Thus, we conclude that the NOTCH1^+^ cells seeded to establish our cultures were likely to be olfactory HBCs rather than possible respiratory epithelial contaminants. The cultured cells consistently formed adherent islands with occasional morphological changes consistent with differentiation (Figure 6B). Representative immunocytochemical staining (Figure 6C) showed nearly homogenous expression of HBC markers KRT5 and TP63. Passaged cultures were further characterized using scRNA-Seq (Figure 6, D and E). Integrating and visualizing the scRNA-Seq data from the cultured sample with scRNA-Seq data sets from 3 acutely harvested human biopsy samples confirmed that the cultures (at passage 6) expressed HBC markers (KRT5, TP63, EGFR) but *not* the respiratory marker SERPINB3 (Figure 6D) and clustered generally with the olfactory HBCs from acute biopsies in UMAP plots (Figure 6E), indicating transcriptional profile similarity to the in vivo HBCs.

We next used this culture model to test the effects of TNF-α on HBC gene expression. Cells were cultured in either HBC growth medium with 0.5 ng/mL TNF-α or HBC growth medium alone for 72 hours (Figure 6F). Quantitative reverse-transcriptase PCR (RT-qPCR) assays showed no change for HBC subset markers identified in our initial scRNA-Seq; however, TP63 levels were increased (Figure 6F). TNF-α–treated HBCs exhibited more than a 2-fold increase in TP63 compared with the HBCs cultured in growth medium alone (*P* = 0.04, *t* test, *n* = 3 replicates), suggesting that an effect of acute TNF-α exposure may promote HBC quiescence, as opposed to differentiation, since TP63 acts to hold HBCs dormant. The culture model will, therefore, provide a means to further assess mechanisms affecting human HBC growth and potential pharmacologic agents.

## Discussion

**Figure 7:**
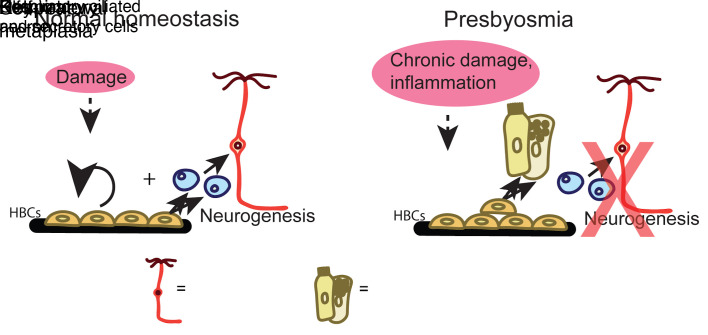
A model for presbyosmic stem cell dysfunction. Aging-related damage promotes an inflammatory milieu in which olfactory HBC alterations result in decreased neurogenic activity. Histologic findings include reactive or layered HBCs along with a respiratory-like metaplasia, which may emerge from altered basal cell differentiation or from repopulation of the surface epithelium from Bowman’s gland/duct progenitors (69) (not depicted).

We provide here a single-cell analysis of olfactory mucosa from presbyosmic and normosmic human subjects. A failure of olfactory neuroepithelial maintenance or homeostasis with aging is thought to account for presbyosmia despite the OE’s robust regenerative capacity. Our results indicate that a loss of stem cells does *not* explain the histologic picture of neurogenic exhaustion that is seen in the aging human OE. Rather, we identify transcriptional alterations in HBCs, the olfactory reserve stem cells, consistent with a model in which aging-related damage and inflammation result in stem cell dysfunction (Figure 7).

As a barrier epithelium, the immune defense functions of the nasal mucosa are well described, especially in the setting of chronic disease states, such as sinusitis or polyposis, including innate and adaptive responses (54, 55). How nasal immune signaling interacts specifically with the highly specialized olfactory neuroepithelial cell populations, especially the neurogenic basal cells, has only recently begun to be understood, largely in rodent models (25). It is evident that the OE must balance the challenges of a typical barrier epithelium with a chemosensory function requiring maintenance of intact populations of olfactory receptor neurons and their replacement following ongoing turnover or severe damage. Unlike mouse models, the human OE must do so for many decades to avoid an aging-related loss of function, i.e., presbyosmia.

Drawing on other self-renewing epithelia, such as the skin or intestinal crypts, it is clear that tissue-resident adult stem cell populations utilize similar regulatory signals, yet hold a remarkable ability to redirect their function or behavior in response to patterns of tissue injury (56, 57). In rodent OE, the GBCs proliferate and handle the “normal” replacement of olfactory neurons as needed, while the typically quiescent HBCs are activated by more severe damage, including barrier disruption that occurs when the sustentacular population is lost (15, 16, 18, 58). Despite these 2 categories of progenitor cells, a mouse model of chronic local cytokine overexpression in the OE fails to reconstitute the neuroepithelium, resulting in HBC populations remaining abnormally dormant in the setting of neuronal and sustentacular cell loss (25, 59). Mechanistically, TNF-α receptors on OE cells appear to mediate cytokine signaling and act via the RelA and NF-κB pathways in basal cells (24). Whether human conditions such as presbyosmia may involve inflammation-mediated stem cell dysfunction has not been determined.

Our results provide the first report, to our knowledge, combining a clinical evaluation of human subjects with presbyosmia and olfactory biopsy sampling utilizing single-cell transcriptional profiling. The variability in successful capture of intact olfactory neuron populations due to respiratory-like metaplasia has been well described in aging human olfactory samples, suggesting the neurogenic exhaustion hypothesis (22). Nonetheless, the present approach is well suited to exploring transcriptional alterations among the cell populations present in control versus presbyosmic samples. Our initial analysis of data sets suggested potential differences among HBCs in presbyosmic versus control groups, while other populations were more homogenous, motivating our further focus on the stem cell analyses. We considered various potential findings that might drive neurogenic exhaustion or respiratory-like metaplasia: (a) stem cells might be sparse or absent; (b) only respiratory-type stem or progenitors might be identifiable; or (c) olfactory stem cells might remain present, but exhibit molecular alterations unique to the presbyosmic samples. Our results support the last model. Focusing attention on the HBCs, or reserve stem cells, we identified heterogeneity among this population not, to our knowledge, previously described (Figure 2). Our data sets provide a basis for ongoing efforts aimed at identifying the roles of HBC subset–specific transcripts, such as CTSV or SPINK5. The inflammatory-response phenotype identified in presbyosmic HBCs appears consistent with aging-related tissue alterations that may drive an immune-defense HBC response, preventing their normal neurogenic program (Figure 7). Finally, our description of a human olfactory HBC culture model will provide a platform to test in vitro modulation of HBC signaling for therapeutic potential.

Limitations of this study include the restriction of our biopsy samples to the peripheral olfactory apparatus, which is the routinely accessible portion of this sensory system. We do not exclude the possibility that presbyosmia is a heterogenous condition that may, in some subjects, be due to central sites of pathology, such as the olfactory bulbs or cortex. The analysis of olfactory mucosa biopsies is not a complete survey of the olfactory cleft, but represents a sampling of the peripheral olfactory organ. Variability in the extent and continuity of olfactory versus respiratory surface epithelium is an unavoidable reality; therefore, we avoid here direct comparisons of specific cell-type yields among samples and instead focus on comparisons of the transcriptional profiles of captured cell populations. Efforts to acquire additional human biopsies from subjects with known olfactory function specifically for immunohistochemical, RNA in situ hybridization, or spatial transcriptomic analyses will be a focus of future investigations into human olfactory pathophysiology.

In summary, approximately 12% of US adults are estimated to have olfactory dysfunction (60), and prevalence increases dramatically over age 65 (9, 10). There are currently over 54 million US residents age 65 or older (US census data), suggesting that presbyosmia affects millions of people. Currently, there are no specific treatments available for sensorineural olfactory disorders, including presbyosmia. The findings and data sets provided here offer insights into the cellular and molecular changes associated with aging-related olfactory loss and should provide a resource for ongoing study. Our conclusions suggest that olfactory stem cells may be a logical therapeutic target for certain sensorineural olfactory disorders. For these findings to be applied to the clinic, preclinical testing of reagents targeting pathways of interest in OE stem cell cultures or animal models may facilitate pilot clinical trials.

## Methods

### Study design.

We performed a prospective analysis of presbyosmic or control normosmic subjects to obtain nasal mucosal biopsies for analysis. No statistical method was used to predetermine sample size; rather, we followed current best practices for scRNA-Seq studies and, guided by goals for rare cell type identification, expected read depth, and identification of DE genes for specific clusters, utilized 3 subjects in each cohort (61). Tissue samples from a single patient were processed individually, as was a single-cell culture sample for comparison. Inclusion and exclusion criteria are discussed below, based on olfactory function scores. The research objective was to identify molecular alterations in olfactory cell populations of presbyosmics. Our initial hypothesis was that molecular alterations consistent with stem cell dysfunction would be identified. Following initiation of data analysis, our hypotheses were expanded to focus also on immune cells and immune-induced alterations in stem cells. Subjects were identified from an academic rhinology practice, without randomization, and blinding was not feasible.

### Sample acquisition and preparation.

Olfactory testing was performed using a validated assessment tool, the SIT. Biopsies of the septum from the olfactory cleft region were obtained by sinus surgeons in the Department of Head and Neck Surgery & Communication Sciences at Duke University School of Medicine via an IRB-approved protocol. Tissue was obtained from patients consented for transnasal endoscopic surgery to access the pituitary or anterior skull base. Samples for scRNA-Seq were selected according to the inclusion criteria as patients presented for routine clinical care. Inclusion criteria included SIT scores of 34 or more for the control group and SIT scores of less than 30 and age greater than 65 years for the presbyosmic group. Subjects were excluded who had evidence of acute nasal or sinus inflammation or obstruction on preoperative endoscopy and imaging. Mucosa was carefully excised from portions of the olfactory cleft along the superior nasal septum or adjacent superior medial vertical lamella of the superior turbinate, uninvolved with any pathology. Immediately after removal, samples were held on ice in HBSS with 0.1% BSA and transported to the laboratory. Under a dissecting microscope, any bone or deep stroma was trimmed away from the epithelium and underlying lamina propria. A small portion of some specimens was sharply trimmed and fixed in 4% paraformaldehyde in PBS, cryoprotected, and then snap-frozen in optimal cutting temperature medium, to be cryosectioned for histology. The remaining specimen was enzymatically dissociated using collagenase type I, dispase, and DNase for approximately 30 minutes. Next, papain was added for 10 minutes, followed by 0.125% trypsin for 1 to 3 minutes, as we have reported (19). Cells were filtered through a 70 μm strainer, pelleted, and washed, and then treated with an erythrocyte lysis buffer and washed again. The cells were then resuspended in PBS with 0.1% BSA and 0.2% anticlumping agent (Invitrogen) and immediately processed for scRNA-Seq using the Chromium (10x Genomics) platform. Fresh human tissue samples used to generate scRNA-Seq data were exhausted in the experimental process with the exception of 1 sample, which was exhausted by use for cell culture (described below).

### Single-cell sequencing.

scRNA-Seq was performed using the Chromium (10x Genomics) platform either through the Duke Molecular Genomics Core or in house. Single-cell suspensions were counted using a hemocytometer, ensuring greater than 80% viability by Trypan dye exclusion, and adjusted to approximately 1000 cells μl^–1^. Samples were run using the Chromium Single Cell 3′ Paired-End, Dual-Index Library & Gel Bead Kit, version 3.1 (10x Genomics). The manufacturer’s protocol was used with a target capture of 5000 to 10,000 cells. Each sample was processed on an independent Chromium Single Cell G Chip; 3′ gene expression libraries were dual-index sequenced using NextSeq 150 bp (Illumina) flow cells by the Duke Center for Genomic and Computational Biology Core Facility.

### scRNA-Seq analysis.

Raw base call files were analyzed using Cell Ranger, version 4.0.0, using the Duke Cluster Computing Platform. The mkfastq command was used to generate FASTQ files, and the counts command was used to generate raw gene-barcode matrices aligned to the GRCh38-2020-A Ensembl 98 human genome. The data from 6 samples were analyzed in R, version 4.1.0, using the Seurat package, version 4.0.3. For quality control, filtering was conducted by (a) removing cells that expressed fewer than 100 genes, (b) removing cells that had mitochondrial gene content of more than 10%, or (c) removing possible homotypic doublets (>8000 genes) (62). In addition, to combat ambient RNA contamination from cell lysis during the sample dissociation and processing steps, we utilized the SoupX R package for background correction of each individual sample prior to data set integration (63). Samples were normalized, scaled, and integrated as we have described (19). Data for all 6 samples, totaling 36,091 cells, were combined using the standard Seurat integration workflow for normalization and batch correction (64). We chose to use 50 principal components based on results from analysis using elbow plots of SD. Clustering was conducted using the FindNeighbors() and FindClusters() functions with a resolution parameter set to 1.0. The resulting 38 Louvain clusters were visualized in a 2D UMAP representation and were annotated as we have described (19). For further analysis of cell types of interest, the subset() command was used. A new analysis on each subset was performed using the FindVariableFeatures(), ScaleData(), RunPCA(), and RunUMAP() functions. New UMAP plots were generated for each subpopulation, along with FeaturePlots or violin plots for specific gene expression visualization. DE between control and experimental groups was identified using the FindMarkers() command, using *P* < 0.05 and log fold change of greater than 0.60 for significance, with manual inspection and removal of uninformative transcripts such as XIST or HBB from further analysis. To visualize differentially expressed genes between groups, volcano plots were generated using the ggplot2 R package to plot log fold change.

### GSEA.

The top 50 enriched genes or all genes with a *P* value of less than 0.05 from the FindMarkers() function were input into the ToppGene suite (65) for GO and biological pathway annotation, with Bonferroni’s correction for significance testing set at *P* < 0.05.

### Receptor-ligand interactions analysis.

R implementation of NicheNet intercellular communication analysis was performed by defining “receiver” cell genes as those differentially expressed between presbyosmic HBCs and control HBCs. Sender cell populations were defined as NK, NKT, and CD8^+^ T cells from presbyosmic patients. Parameters and significance thresholds for receptors, ligands, and target genes were set as recommended (43). Highly validated receptor-ligand pairs were determined by using the protein-protein interaction (PPI) prediction database assembled in NicheNet (https://github.com/saeyslab/nichenetr; commit ID Ba7238f).

### Immunohistochemistry.

Cryosections were prepared from nasal epithelium biopsies as described (19). Briefly, after PBS rinse and any required pretreatments, tissue sections were incubated in blocking solution with 5% donkey serum and 0.1% Triton X-100 for 30 minutes at room temperature. Primary antibodies (Supplemental Table 1) were diluted in the blocking solution and incubated overnight in a humidified chamber at 4°C. Detection by species-specific fluorescent-conjugated secondary antibodies (Jackson ImmunoResearch) was performed at room temperature for 45 minutes. Sections were counterstained with DAPI, and coverslips were mounted using Vectashield (Vector Labs) for imaging, using a Leica DMi8 microscope system (Leica Microsystems). Images were processed using ImageJ software, version 2.1.0/1.53c (NIH). Scale bars were applied directly from the Leica acquisition software metadata in ImageJ Tools.

### Human olfactory HBC cultures.

After loading the appropriate volume of cell suspension to the 10× chromium controller from control sample 3, the remaining cell suspension was used for cell culture. To enrich for olfactory HBCs, which have been found to utilize Notch signaling (18), the sample was sorted using an APC-conjugated antibody to NOTCH1 (eBiosciences, catalog 17-5765-80) followed by APC magnetic selection using the EasySep Kit (STEMCELL Technologies) per the manufacturer’s instructions and plated in PneumaCult medium with a SMAD inhibitor, as we have described for mouse basal cell expansion (66). Cells were plated on 5% laminin, as described for unsorted rat basal cells (67), at 50,000 cells per well of a 6-well plate and incubated in full growth medium: PneumaCult-Ex medium (STEMCELL Technologies), glutamax supplement (Invitrogen), penicillin-streptomycin (Invitrogen), 10 μM Y27632, 20 ng/ml EGF, and 10 μM A83-01 (all from STEMCELL Technologies). Y27 was removed from medium 24 hours after initial plating. After the initial medium change, the medium was exchanged every 48 hours. Importantly, the scRNA-Seq analysis of the biopsy specimen from which this culture was established revealed almost exclusively olfactory rather than respiratory populations (see Figure 1, C and D), suggesting minimal potential culture contamination by respiratory basal cells. Cultures were passaged splitting at 1:3 with brief trypsinization when –80% confluent. Cells were frozen in 90% FBS (Invitrogen) with 10% dimethyl sulfoxide and stored in liquid nitrogen. Cell phenotype was characterized as described below.

### TNF-α treatment.

Passage 4 human HBCs, cultured as described above, were plated at a concentration of 20,000 cells per well of a laminin-coated 24-well plate. TNF-α (0.5 ng/mL final concentration in PBS, human recombinant, STEMCELL Technologies) was added to half of the wells, while the other wells received full growth medium only, with vehicle. Medium was changed after 48 hours. After 72 hours, RNA was isolated for analysis. All assays were performed in triplicate.

### RT-qPCR.

Total RNA was isolated using column purification per protocol (Zymo Research Corp.). DNase I on-column digestion was performed. Reverse transcription first-strand cDNA synthesis was performed using Superscript IV (Invitrogen). Validated TaqMan FAM probe primer assays (Invitrogen) were used in reactions performed on a Bio-Rad real-time thermal cycler; probes included CTSV (Hs00426731_m1), SPINK5 (Hs00928570_m1), TP63 (Hs00978344_m1), and GAPDH (Hs02758991_g1). Reactions were performed in triplicate, and at least 3 replicates were tested per condition. Fold-change calculations were performed using the 2^–ΔΔCt^ technique (68), and GAPDH expression was used as a reference.

### Data and materials availability.

Data were deposited in the NCBI’s Gene Expression Omnibus database (GEO GSE184117).

### Statistics.

No statistical method was used to predetermine sample size. For each experiment, tissue samples from a single patient were processed individually. Single-cell suspensions for each sample were processed for scRNA-Seq in an independent chromium chip (10x Genomics). For differential expression analysis in Seurat, the default 2-sided nonparametric Wilcoxon’s rank-sum test was used with Bonferroni’s correction using all genes in the data set. GSEA was performed as described. qPCR comparisons were analyzed by 2-tailed *t* test using GraphPad Prism, version 9. *P* < 0.05 was considered significant.

### Study approval.

All human subject research was performed according to a protocol approved by the IRB at Duke University via surgical informed consent permitting research on surgical specimens that are otherwise discarded. Subjects undergoing endoscopic transnasal procedures to access the pituitary or anterior skull base require removal of small portions of olfactory cleft tissue for access, and this tissue, uninvolved with any pathology, is normally discarded, but permits recovery of olfactory mucosal cells (19) per approved protocol.

## Author contributions

BJG conceived the project. ADO, KI, RAH, DWJ, RG, SAW, and BJG contributed to methodology. ADO, HM, EAM, SAW, and BJG contributed to data visualization. ADO, EAM, and BJG acquired funding. HM and BJG supervised the project. ADO and BJG wrote the original draft of the manuscript. ADO, SAW, KI, RAH, DWJ, RG, EAM, HM, and BJG reviewed and edited the manuscript.

## Supplementary Material

Supplemental data

ICMJE disclosure forms

Supplemental tables 2-6

## Figures and Tables

**Table 1:**
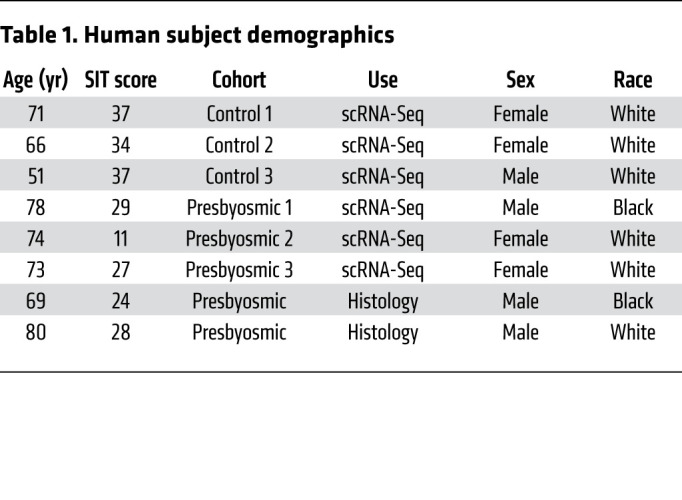
Human subject demographics

## Version 1 01/06/2022

In-Press Preview

## Version 2 02/15/2022

Electronic publication
