# Supplementary material for: Aging-related olfactory loss is associated with olfactory stem cell transcriptional alterations in humans
Source: J Clin Invest. 2022 Feb 15;132(4):e155506. doi: 10.1172/JCI155506 (PMC8843745; doi:10.1172/JCI155506)
Supplement: Supplemental data [file jci-132-155506-s088.pdf]

Supplemental Materials:

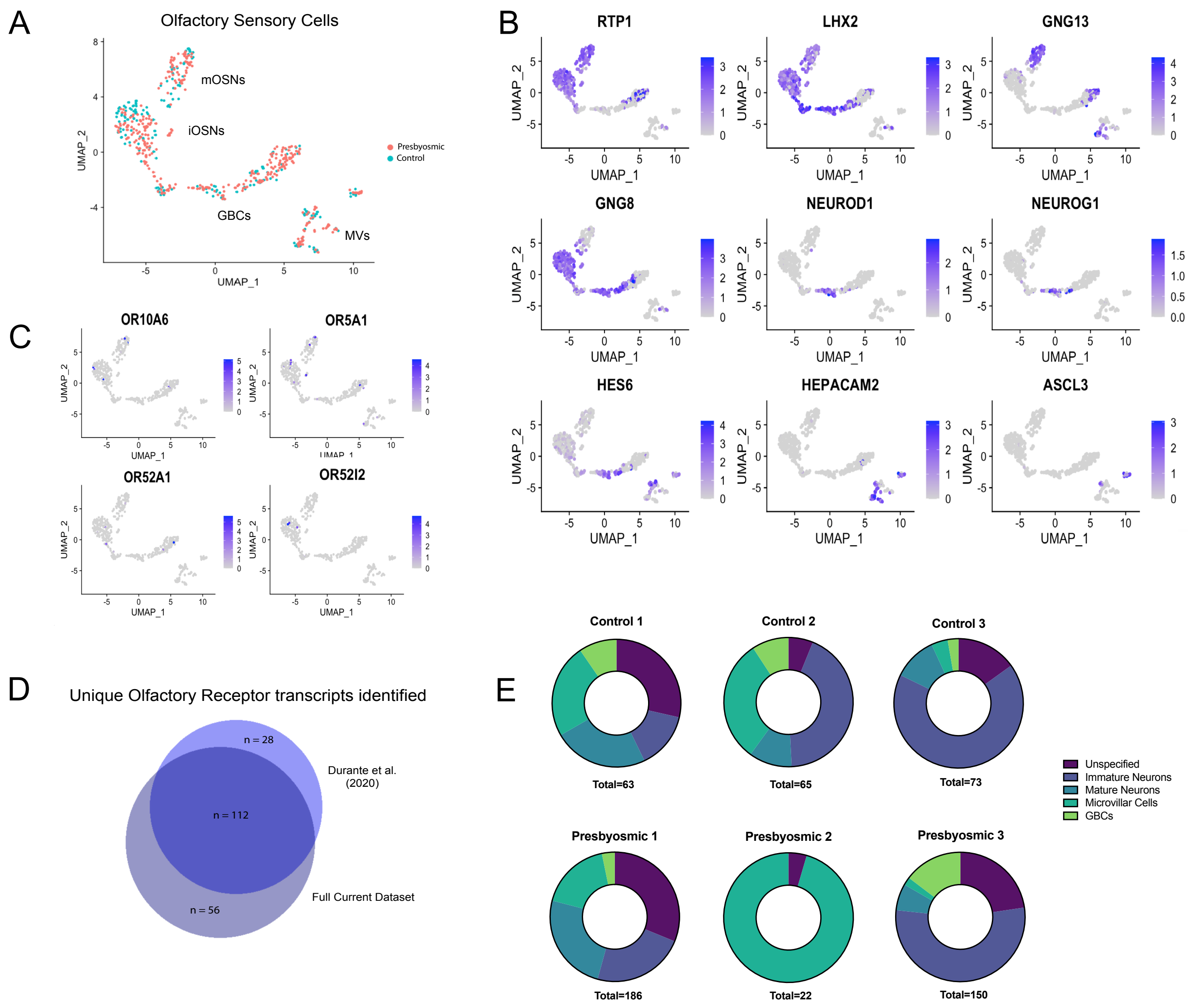

Supplemental Figure 1. Analysis of sensory cell clusters. (A) Sensory cell cluster comprised by mature olfactory neurons (mOSNs), immature olfactory neurons (iOSNs), GBCs and microvillar sensory cells (MVs) was re-plotted as UMAP, visualized by original sample identification. **(B)** FeaturePlot visualization showing selected cell type-specific marker expression among sensory cell populations. RTP1 and LHX2 are expressed in olfactory neurons; GNG13 and GNG8 are expressed by mature or immature neurons, respectively; NEUROD1, NEUROG1 and HES6 are expressed by GBCs; HEPACAM2 and ASCL3 are expressed by

microvillar sensory cells. **(C)** FeaturePlot visualization of selected OR expression. OR10A6 and OR5A1 are Class II ORs; OR52A1 and OR 52I2 are Class I ORs; note scattered expression among neuron cell clusters, as expected. **(D)** Venn diagram depicting the unique ORs identified in our previous scRNA-seq data (19) and the current dataset. We identified 56 additional ORs in the present study, for a total of 196 unique OR transcripts from human biopsies of 10 subjects. See “Supplemental Table 2” spreadsheet for OR list. **(E)** Cell type composition of sensory clusters by sample. Note “Presbyosmic 2”, which lacks olfactory neurons, was anosmic by SIT.

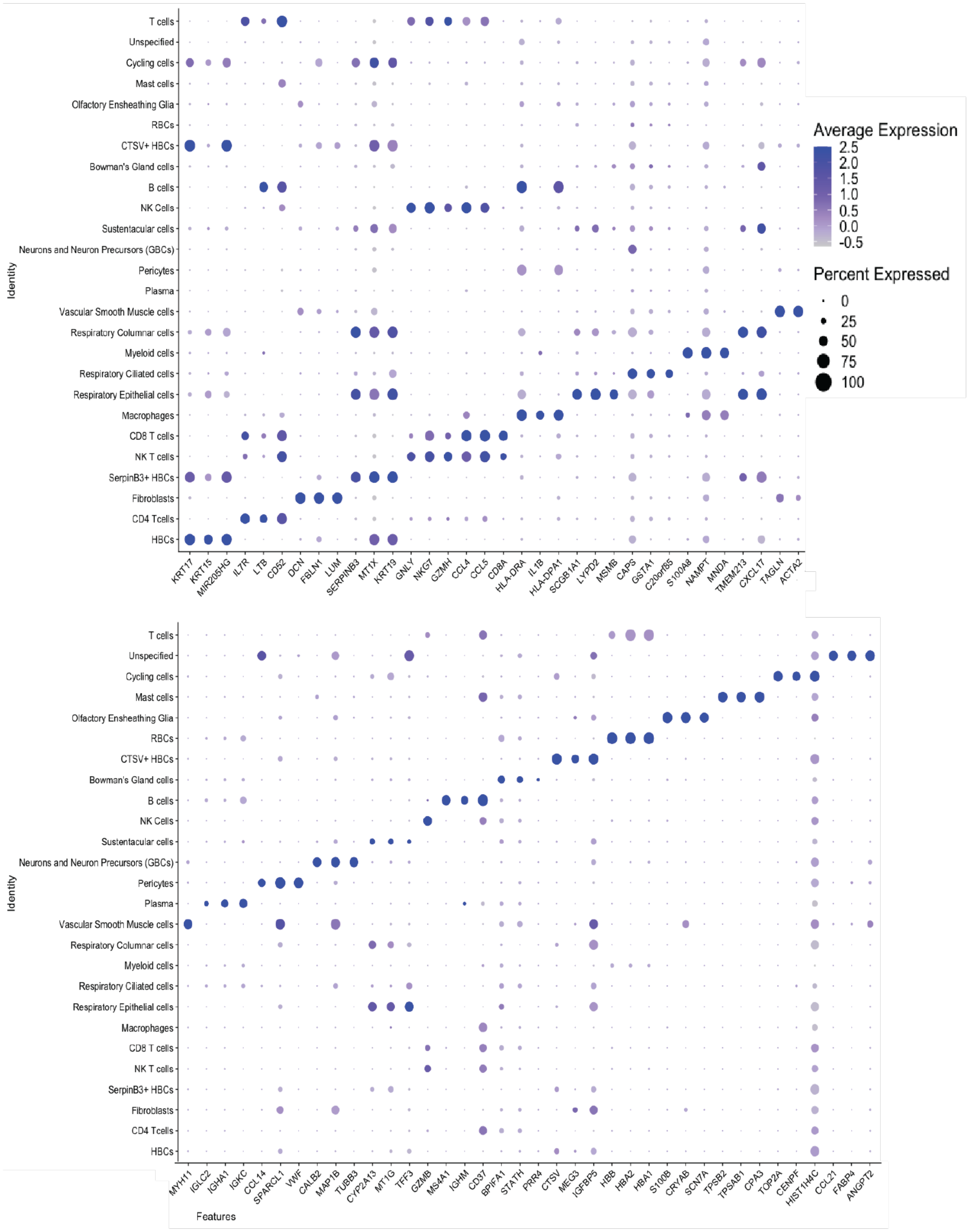

Supplemental Figure 2. DotPlot analysis of integrated sample. Plot depicts unbiased gene expression for top 3 enriched genes per cluster, UMAP plot is shown in Fig. 1D. Cell cluster

identity is indicated on the y-axis; gene names (Features) are indicated on the x-axis. The plot depicts clusters from 36,091 cells, n=6 subjects.

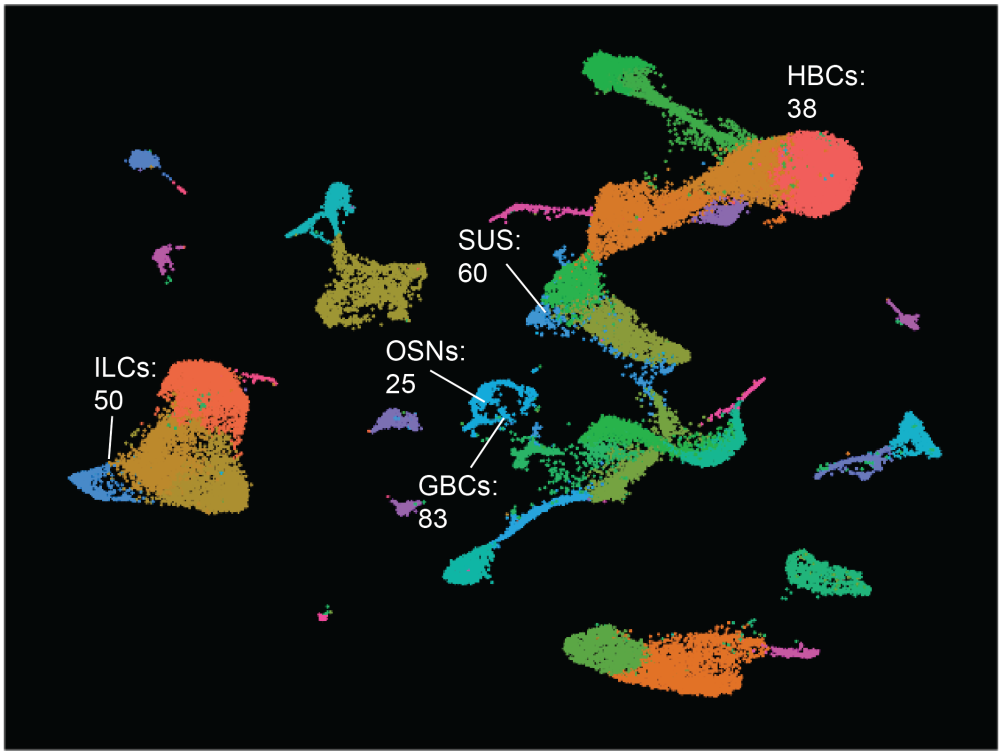

Supplemental Figure 3. Differential expression overview. Number of significant DE genes are listed for cell clusters of interest (p,<0.05 and log fold change >0.6). Sus = sustentacular cells; ILCs = innate lymphoid compartment; OSNs = olfactory sensory neurons; GBCs = globose basal cells; HBC = horizontal basal cells. Analysis of DE gene sets suggested functional importance especially for the HBC and ILC clusters, as described.

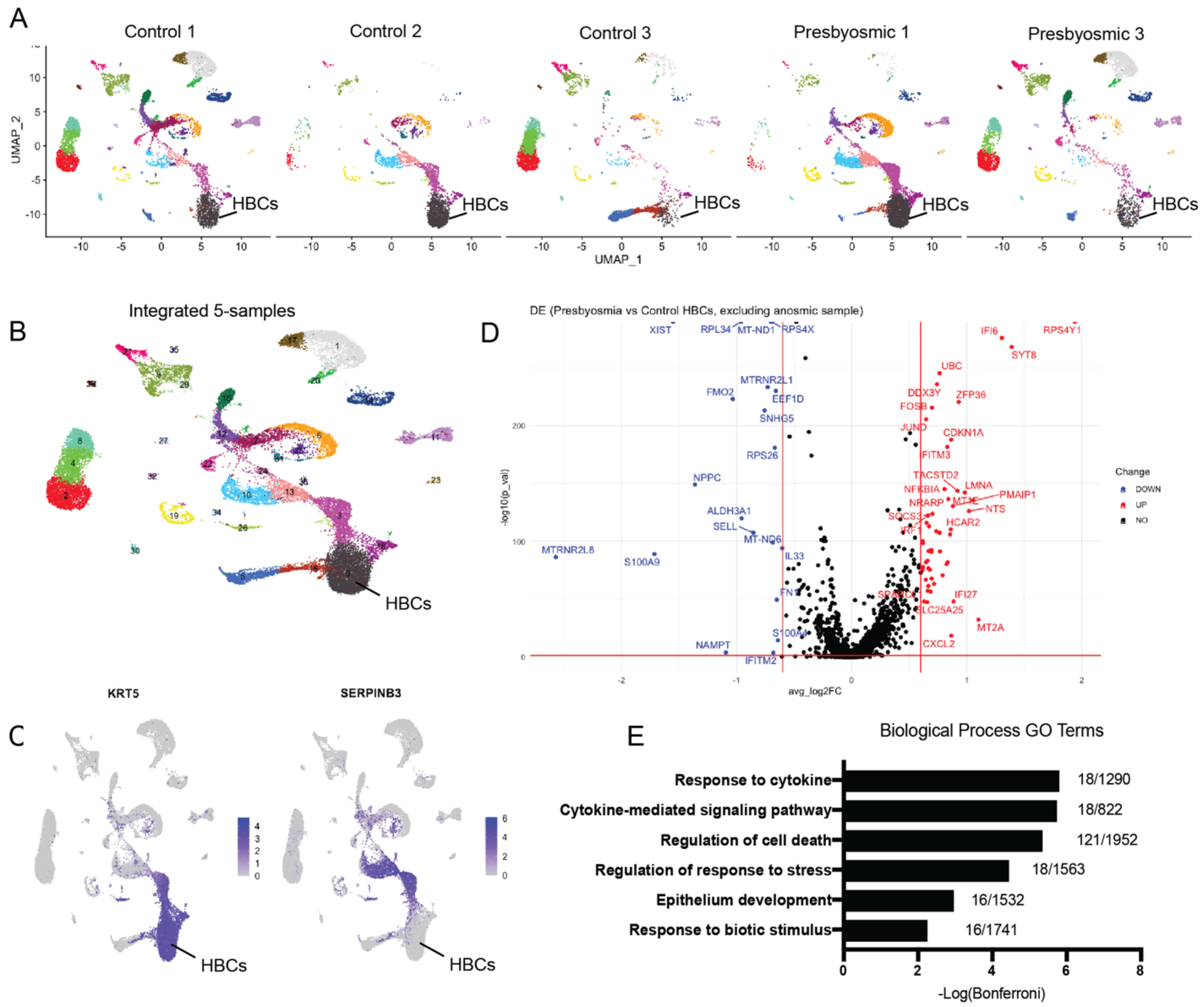

Supplemental Figure 4. Presbyosmic HBC gene expression changes are <u>not</u> driven solely by the single totally anosmic sample. Re-analysis of presbyosmic HBCs excluding the totally anosmic sample yields similar differential gene expression as identified in full analysis, comparing the two hyposmic samples to the three normosmic controls. **(A)** UMAP plots showing the individual 5 samples re-analyzed here. **(B)** Integrated 5-sample UMAP re-projection; the olfactory HBC cluster is annotated. **(C)** FeaturePlots confirm the phenotype of HBC cluster, which is KRT5(+)/SERPINB3(-). **(D)** Volcano plot depicting differential gene expression comparing presbyosmic HBCs from 2 hyposmic samples to the HBCs from 3 normosmic

samples; significantly upregulated ($p < 0.05$ and log fold change >0.60) genes in presbyosmic HBCs are marked (red). **(E)** Selected significant GO terms are shown, confirming that presbyosmic HBCs upregulate inflammatory response gene sets, despite exclusion of the totally anosmic sample. DE = differential expression; GO = gene ontology.

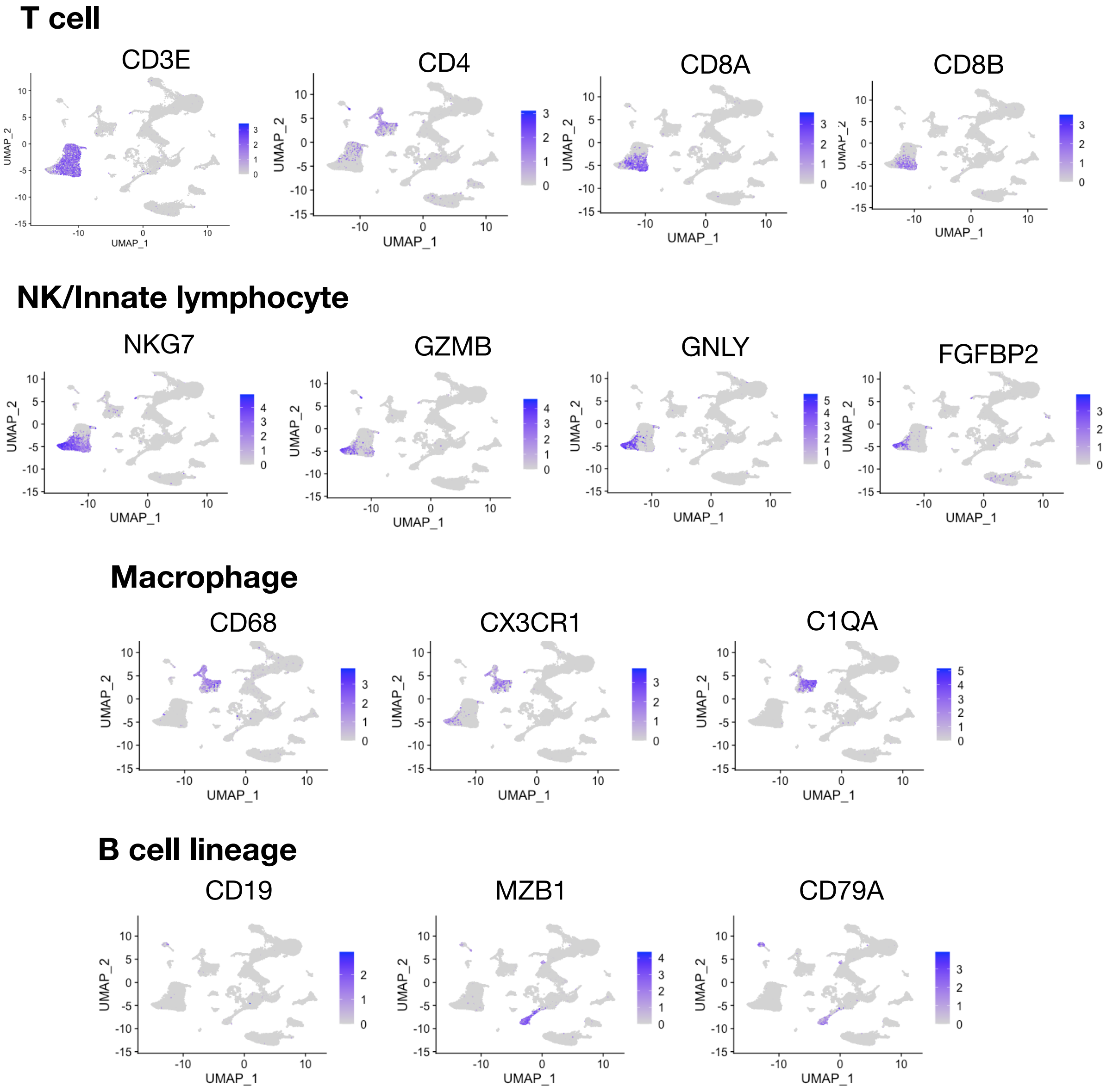

Supplemental Figure 5. Analysis of immune cell clusters. FeaturePlots showing indicated gene expression across integrated UMAP projections of combined data sets from normosmic and presbyosmic samples. Markers expressed by indicated immune cell phenotypes are shown, including T cells, NK/Innate lymphoid compartment (ILC) cells, Macrophages, and B cell lineages.

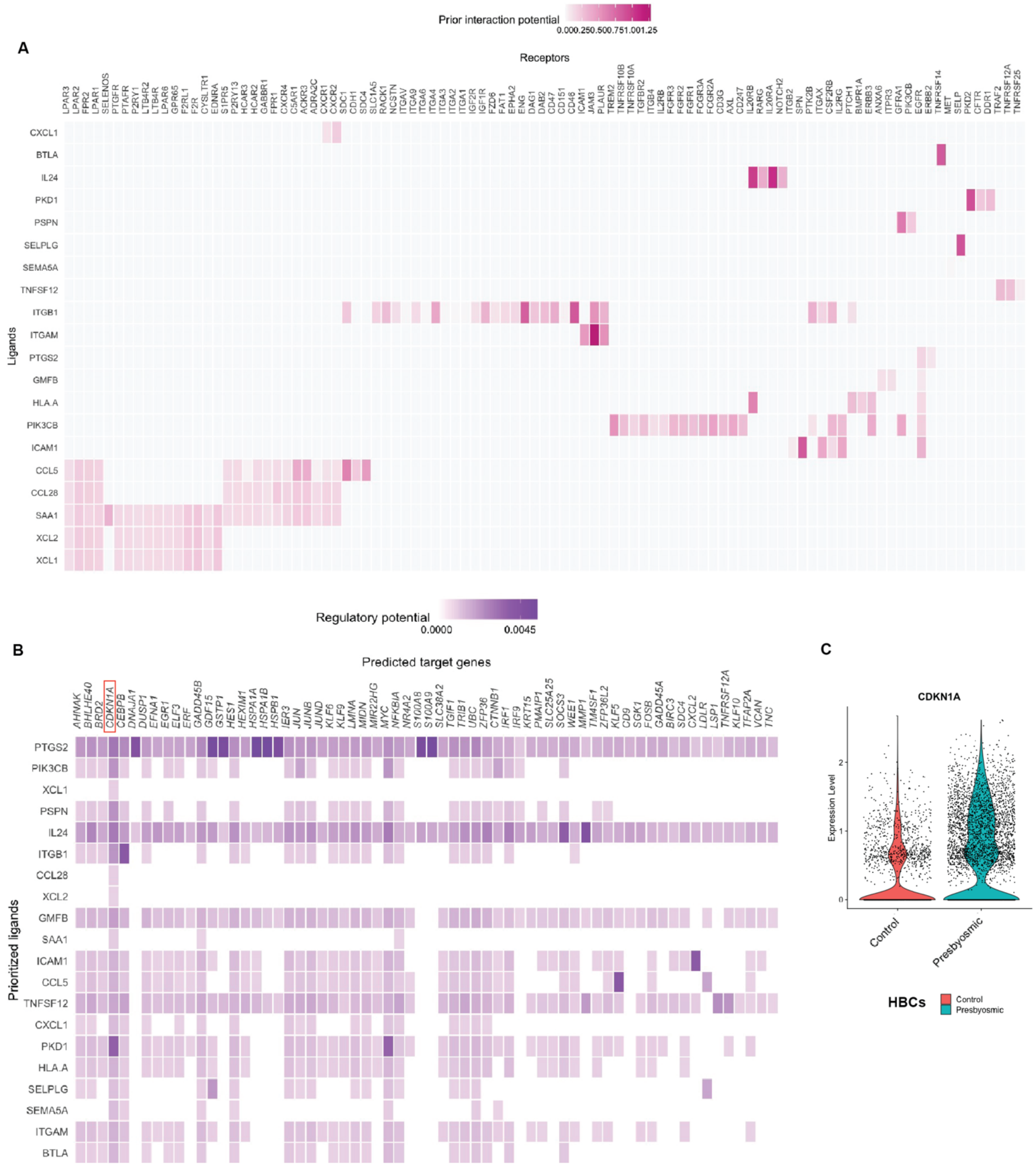

Supplementary Figure 6. Complete List of Presbyosmic Lymphocyte Ligands and HBC Targets. (A) Complete list of presbyosmic NK, NKT, and CD8+ T cell ligands and presbyosmic HBC receptors displayed **in Fig. 5A**, with a lower threshold for receptor-ligand interaction

probability. **(B)** All predicted presbyosmic HBC target genes, displayed as in **Fig. 5B**. **(C)** CDKN1A, a downstream target gene of all ligands plotted in (B) and Fig. 5B, is upregulated in presbyosmic HBCs.

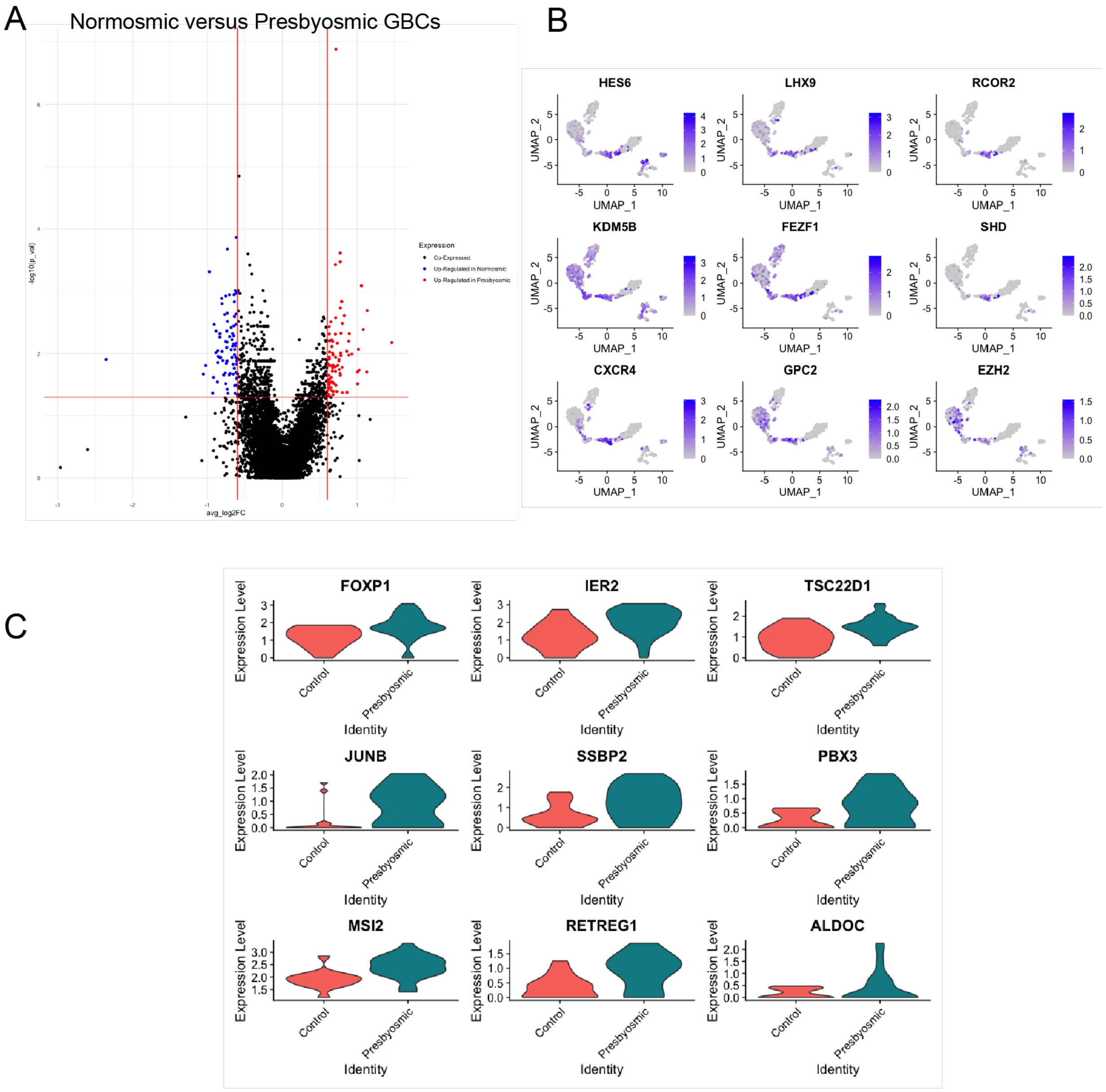

Supplemental Figure 7. Globose basal cell (GBC) comparison, presbyosmic versus control samples. (A) Volcano plot depicting differential gene expression; 83 genes are upregulated ($p < 0.05$ and log fold change >0.60) in presbyosmic GBCs (red). **(B)** FeaturePlots showing indicated gene expression across the sensory cell subset plot (see Supplemental Fig. 1A for cluster annotation); although the GBC population is limited, high percentages of cells in the GBC cluster express typical GBC genes such as HES6, LHX9, and RCOR2. **(C)** Violin plots

depicting differential expression of selected genes between presbyosmic and control GBCs; inflammatory and early response genes are among upregulated DE genes in presbyosmic cells, including IER2, CXCR4 and JUNB. However, significant GO terms for the set of upregulated presbyosmic GBC genes was not clearly informative, suggesting that the GBC phenotype is not substantially altered.

Supplemental Table 1. Primary Antibodies

Target	Host Species	Source	Catalog Number	RRID	Dilution
CK5	Rabbit	Abcam	ab52635	AB_869890	1:1000
DCX	Rabbit	Cell Signaling	4604	AB_561007	1:200
SERPINB3	Mouse	Abcam	ab180396	AB_2892671	1:100
SERPINB3	Rabbit	Sigma-Aldrich	HPA055992	AB_2682998	1:100
TUJ1	Mouse	Biolegend	801201	AB_2313773	1:500
TUBB4	Mouse	Sigma-Aldrich	T6793	AB_477585	1:150
TP63	Mouse	Santa Cruz Biotechnology	Sc-5301	AB_628093	1:500

OTHER:

Supplemental Tables combined (.xls file). List of olfactory receptor transcripts; DE gene lists.
